# Functionally Gradient Macroporous Polymers: Emulsion Templating Offers Control over Density, Pore Morphology, and Composition

**DOI:** 10.1021/acsapm.4c00261

**Published:** 2024-04-24

**Authors:** Yufeng Xu, Le Tang, Chanokporn Nok-iangthong, Markus Wagner, Georg Baumann, Florian Feist, Alexander Bismarck, Qixiang Jiang

**Affiliations:** † Institute of Material Chemistry and Research, Faculty of Chemistry, University of Vienna, Währinger Strasse 42, 1090 Vienna, Austria; ‡ Institute for Vehicle Safety, Graz University of Technology, Inffeldgasse 13 VI, 8010 Graz, Austria; § Department of Chemical Engineering, Imperial College London, South Kensington Campus, London SW7 2AZ, U.K.

**Keywords:** emulsion templating, macroporous polymers, functionally gradient foams, impact resistance, impact protection

## Abstract

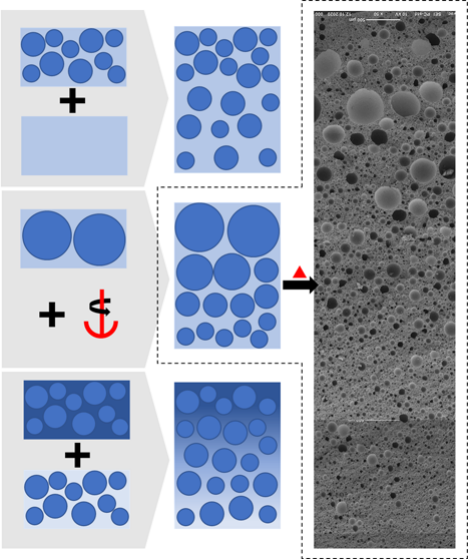

Gradient macroporous polymers were produced by polymerization of emulsion templates comprising a continuous monomer phase and an internal aqueous template phase. To produce macroporous polymers with gradient composition, pore size, and foam density, we varied the template formulation, droplet size, and internal phase ratio of emulsion templates continuously and stacked those prior to polymerization. Using the outlined approach, it is possible to vary one property along the resulting macroporous polymer while retaining the other properties. The elastic moduli and crush strengths change along the gradient of the macroporous polymers; their mechanical properties are dominated by those of the weakest layers in the gradient. Macroporous polymers with gradient chemical composition and thus stiffness provide both high impact load and energy adsorption, rendering the gradient foam suitable for impact protective applications. We show that dual-dispensing and simultaneous blending of two different emulsion formulations in various ratios results in a fine, bidirectional change of the template composition, enabling the production of true gradient macroporous polymers with a high degree of design freedom.

## Introduction

Porous structures with gradient pore morphologies can be found in many natural biological materials such as bones and bamboo. Lightweight gradient porous materials possess excellent mechanical properties.^1,2^ Such biological materials inspired researchers to develop functionally graded foams, such as porous polymers with gradient pore morphologies,^3,4^ focusing primarily on impact adsorbing materials.^5^ Simulation work suggested that porous polymers with a gradient foam density are advantageous at adsorbing low impact energy and reducing accelerating impact duration and are, therefore, a potential candidate for helmet liners.^6,7^ Porous polymers with gradient microstructures have also been explored for separation applications. One good example is the use of asymmetric polymer membranes, e.g., produced by solvent-induced phase separation, for liquid–liquid separation.^8^ Using macroporous polymers as micromixers for continuous chemical reaction, mixing and emulsification has been reported;^9,10^ we anticipate that macroporous polymers with gradient pore structures may be superior to the mixers with a homogeneous pore structure when the physical properties, such as viscosity, of the reagents change along these processes.

A straightforward method to produce gradient cellular structures is additive manufacturing,^11−14^ for example, fused deposition modeling. A cellular structure with gradient foam density can be created by depositing layers with a gradually changing infilling degree. A few methods to create gradient porous microstructures have been disclosed; Torres-Sanchez et al.^15,16^ produced gradient polymer foams by sonication of a mixture of diisocyanate, polyol, and a blowing agent along the polymerization and foaming process. Ultrasonication affected the convective mass transfer during the foaming process and the diffusion of the blowing agent. Polyurethane foams with different porosity gradients were prepared by controlling the intensity of the acoustic pressure and changing the location of the acoustic field in the reaction mixture. Induced phase separation techniques, including nonsolvent-induced phase separation (NIPS),^17,18^ thermally induced phase separation (TIPS),^18−21^ polymerization-induced phase separation (PIPS),^12^ and vapor-induced phase separation (VIPS),^22^ are also used to produce sheet-like porous polymers with controlled gradient pore morphologies. Indeed, these methods allow for the generation of gradient structures by controlling the processing parameters, e.g., temperature or concentration gradient and orientation of the phase separation front; the gradient tendency (e.g., linear, nonlinear, etc.) and resolution can hardly be tailored. An alternative strategy to produce gradient foams is to layer a series of formulations containing particulate templates, e.g., ice^23^ or gelatin^24^ particles, with various sizes and loading. After the solidification of the polymer phase and the removal of the templating phase, porous polymers with a templated gradient structure were produced. Despite the usefulness of the method in producing porous polymers with well-defined gradient pore structures, the preparation process is labor intensive as each layer requires an individual formulation limiting the applicability of the method to produce true gradient foams.

Emulsion templating was first reported in 1962 by Bartl and von Bonin^25^ as a method to produce porous polymers with controllable morphologies. An emulsion consisting of a continuous monomer phase and an internal dispersed phase is prepared; the continuous phase of the emulsion is polymerized, followed by the removal of the internal phase.^26,27^ Typically, high internal phase emulsion (HIPE) templates with an internal phase volume ratio greater than 74% are used; the resulting macroporous polymers are therefore called poly(merised)HIPEs. The pore diameters and porosities of polyHIPEs can be adjusted by controlling the droplet diameter and internal phase volume ratio of the emulsion template, respectively.^26,27^ More importantly, emulsions are viscous liquids that can be shaped using casting, printing, and mixing, prior to solidification. These advantages of emulsion templates render them candidates for the production of gradient macroporous polymers. Elsing et al.^2^ prepared water-in-styrene/divinylbenzene emulsion templates using a microfluidic system. Water droplets were injected into the continuous monomer phase in a controlled manner, resulting after polymerization in macroporous polymers with a well-controlled gradient pore size and foam density. The problem associated with microfluidics is their extremely low production rate. Ahmed et al.^28^ reported the preparation of gradient porous poly(vinyl alcohol) and polyacrylamide; the corresponding emulsion templates were centrifuged to create a gradient distribution of emulsion droplets prior to their solidification. The inadequacy of the method is that only a unidirectional emulsion droplet gradient distribution can be realized by centrifugation. To prepare a porous polymer with a single-step gradient, Langford et al.^29^ cast two layers of different emulsion templates in one mold. Because of their high viscosity, the emulsion templates did not mix, resulting after curing in bilayer polyHIPEs with a distinguished interfacial regime. By using different emulsion templates, the authors also produced bilayer polyHIPEs with varying physical and chemical properties. Jurjevec et al.^30^ produced o/w emulsion templates containing monomers with anionic and cationic functional groups. The emulsion templates were stacked prior to polymerization, resulting in macroporous hydrogels containing anionic and cationic functional groups, respectively, located in two hydrogel layers, facilitating the adsorption of positively and negatively charged dyes, respectively. McKenzie et al.^31^ reported the synthesis of elastic poly(dimethylsiloxane) (PDMS) with bidirectional gradient porosity (75 → 70 → 60 → 70 → 75%) by horizontally placing and polymerizing emulsions with different internal phase ratios. Barkan-Öztürk et al.^14^ prepared emulsion templates by coinjection of a continuous and internal phase into a micromixer. By changing the injection rate and back pressure, emulsion templates with adjustable gradient internal phase volume ratios ranging from 74 to 89% and droplet diameters were prepared continuously. The emulsions were 3D printed and in situ ultraviolet (UV) polymerized to prepare gradient polyHIPEs. Kleger et al.^32^ reported the fabrication of gradient porous materials by stereolithographic printing of stable photocurable Pickering emulsions. The gradient emulsion was produced by layer-by-layer printing of emulsion inks having a water content of 0 to 50 vol %. After UV curing and calcination, a porous ceramic with a gradient porosity along the height of the cylinder was created.

The “gradient” concept indicates a desired property varying from A to B. Such a gradient can be realized by selecting two formulations defining properties A and B. Stacking such formulations in certain ratios of A and B will result in a gradient. As such, two emulsion templates, A and B, can be mixed to achieve intermediate properties; stacking and polymerizing these emulsions can result in gradient macroporous polymers with a property profile varying between A and B. This mixing–stacking method needs only two starting formulations as compared to formulation-intensive methods, such as particle templating^23,24^ and some emulsion-templated gradient foams.^31^ Stacking emulsion templates in various sequences could allow much greater design freedom of gradient materials as compared to the process-controlled methods, such as centrifuge-induced gradient emulsion templates.^28^ Comparing the production of gradient emulsion templates using microfluidic systems^2^ with the mixing–stacking method, the latter is advantageous because of its high production rate. We will show that emulsion templating is a facile production method for gradient porous polymers with a chemical, pore size, and porosity gradient. The pore morphologies and densities of the macroporous polymers were determined to verify the control over these properties. The effects of the gradient pore structure and composition on the mechanical and impact properties of the gradient macroporous polymers will be investigated.

## Experimental Section

### Materials

Styrene (St), divinylbenzene (DVB), azobis(isobutyronitrile) (AIBN), 2-ethylhexyl acrylate (EHA), and CaCl_2_·2H_2_O were purchased from Sigma-Aldrich. Polyurethane diacrylate (PUDA) Ebecryl 8402 was kindly supplied by Allnex (Netherlands) and the surfactant Hypermer B246 by Croda (Spain). All chemicals were used as received.

### Preparation of Emulsion Templates

Emulsion templates were prepared in a reaction vessel equipped with a dropping funnel and a glass anchor stirrer connected to an overhead stirrer (IKA RW20 digital). The basic emulsions (E1, E5, E6, E7, and E10) were formulated by adding the internal phase, an aqueous solution of 10 g/L CaCl_2_·2H_2_O, dropwise into the continuous monomer phase while stirring at a speed of 400 rpm. After the addition of the internal phase, the emulsions were further homogenized at the desired stirring speed (Table 1) for 3 min. To formulate emulsion templates with various internal phase ratios, E2–E4 were prepared by diluting E1 with its own continuous phase by mixing at a power input of 100 W for 3 min. E8 and E9 were prepared by mixing E1 and E10 in 2:1 and 1:2 ratios, respectively. For the preparation of E2–E4, E8, and E9, a 450 W hand-held kitchen mixer was used to mix emulsion templates or E1 with its continuous phase. Details of the emulsion compositions are listed in Table 1.

**Table 1:** Detailed Information on Emulsion Composition and Emulsification Method

	continuous phase								
	St^a^	DVB	PUDA	EHA	B246	AIBN	internal phase	2nd stirring speed (rpm)	internal phase ratio^b^
E1	52.2	20.9	10.4	-	15.7	0.8	aq 10 g/L CaCl_2_	1000	80%
E2	E1 diluted with CP^c^ of E1 with a volume ratio of 5:1 by mixing at a power input of 100 W for 3 min								75%
E3	E1 diluted with CP of E1 with a volume ratio of 3:1 by mixing at a power input of 100 W for 3 min								60%
E4	E1 diluted with CP of E1 with a volume ratio of 2:1 by mixing at a power input of 100 W for 3 min								50%
E5	52.2	20.9	10.4	-	15.7	0.8	aq 10 g/L CaCl_2_	2000	80%
E6	52.2	20.9	10.4	-	15.7	0.8	aq 10 g/L CaCl_2_	1500	80%
E7	52.2	20.9	10.4	-	15.7	0.8	aq 10 g/L CaCl_2_	400	80%
E8	E1 mixed with E10 with a volume ratio of 2:1 by mixing at a power input of 100 W for 3 min								80%
E9	E1 mixed with E10 with a volume ratio of 1:2 by mixing at a power input of 100 W for 3 min								80%
E10	-	-	36	60	3	1	aq 10 g/L CaCl_2_	1000	80%

a The volume ratio of St, DVB, PUDA, EHA, B246, and AIBN are provided with respect to the volume of the continuous phase.

b The internal phase volume ratio is with respect to the volume of the emulsion template.

c CP = continuous phase.

### Production of Homogeneous and Gradient Macroporous Polymers

**Scheme 1:**
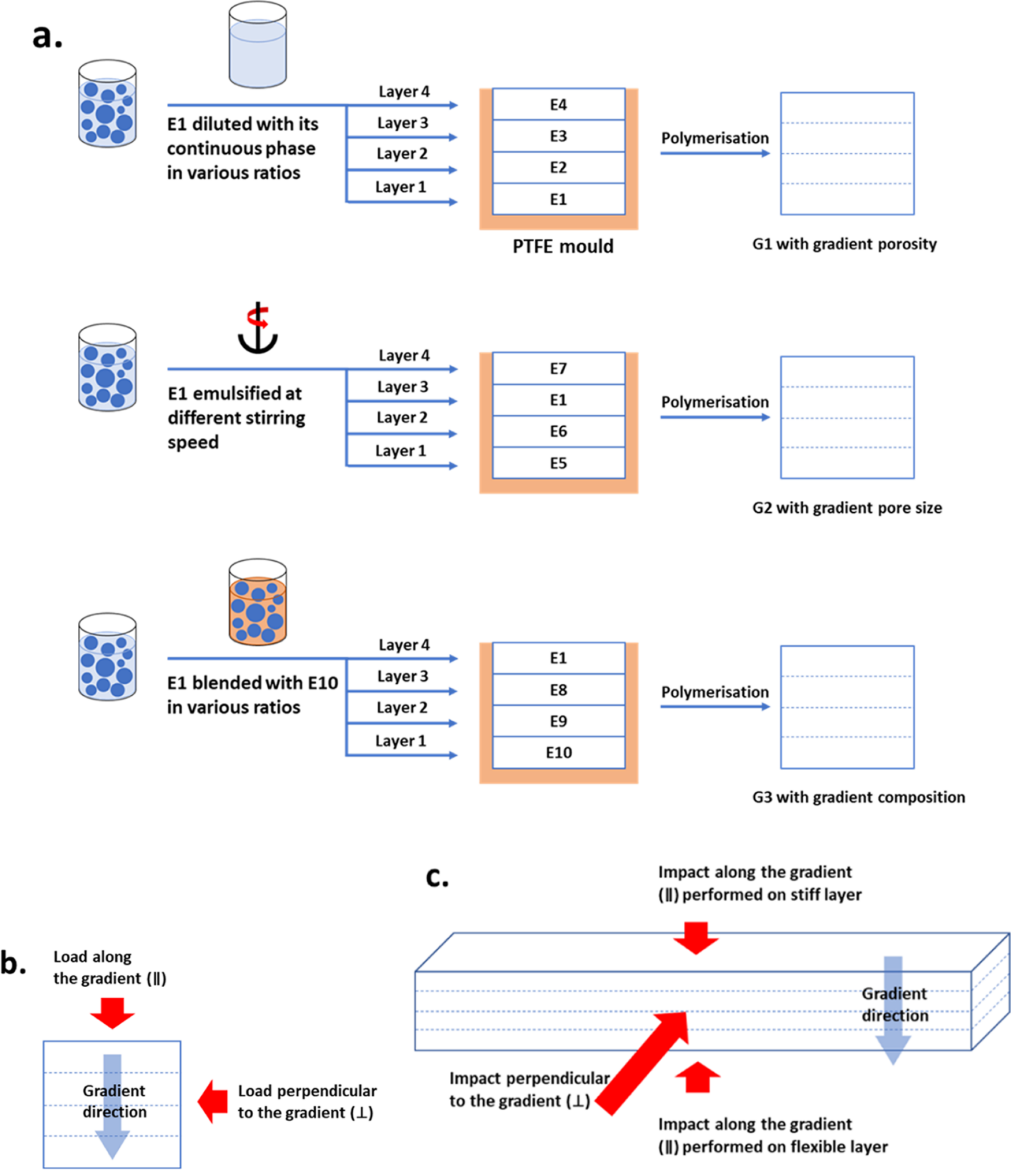
(a) Schematic of the Production Procedure and Composition of Gradient Macroporous Polymers G1–G3. (b) Compression Loading Along (∥) or Perpendicular (⊥) to the Gradient Created in Macroporous Polymers. (c) Impact Loading Along (∥) or Perpendicular (⊥) to the Gradient

To produce emulsion-templated homogeneous macroporous polymers, the emulsion templates (E1–E10) were cast into poly(tetrafluoroethylene) (PTFE) molds with dimensions of 2.5 × 7.5 × 10 and 1.5 × 7.5 × 10 cm^3^. To produce macroporous polymers with gradient porosity, pore size, or polymer compositions, four different emulsion templates were cast on top of each other with the same layer height into the two PTFE molds (Scheme 1a). We cast the emulsions in a sequence from high to low viscosity to avoid interlayer mixing. The molds were closed and placed into an oven to polymerize the continuous emulsion phase at 70 °C overnight. The resulting macroporous polymers were purified in water and ethanol for 24 h, respectively, and dried at 70 °C until they reached constant weight. Homogeneous macroporous polymers are denoted as Sx, while the gradient samples are called G1–G3, where L1–L4 refer to the porous polymer layers from bottom to top.

### Characterization of Macroporous Polymers

The pore morphology of the macroporous polymers was determined from scanning electron micrographs taken using scanning electron microscopy (SEM) (Joel JCM-6000, Joel, Germany). The macroporous polymers were mounted on the SEM stub with a conductive sticker. The fracture surface of the macroporous polymer was coated with gold using a fine coater (JFC-1200, Jeol GMBH, Germany) to ensure electrical conductivity. The surface was observed at a beam energy of 15 kV in secondary electron beam mode. The average pore diameters *d*_p_ and pore throat *d*_pt_ were determined by analyzing the SEM images by using software ImageJ. At least 100 pore and pore throat diameters were measured to obtain average *d*_p_ and *d*_pt_.

The skeletal density ρ_s_ of the macroporous polymers was measured using a helium pycnometer (Accupyc ll 1340, Micromeritics Ltd., Achen, Germany); 0.1 g powder of the polymers was subjected to the measurement. To determine the foam density ρ_f_, the macroporous polymers were cut into specimens with dimensions of 2.5 × 2.5 × 2.5 cm^3^ and weighed; their density was calculated by the mass divided by volume. The gradient macroporous polymers were also cut into four layers of macroporous polymers, and ρ_f_ of each layer was determined. The porosity *P* of the macroporous polymers was calculated as follows
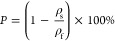
1

### Mechanical Properties of Macroporous Polymers

Compression tests were conducted using a universal dual-column test frame (Instron 5969, Instron GmbH, Germany) equipped with a 50 kN load cell. The macroporous polymers were cut into specimens with dimensions of 2.5 × 2.5 × 2.5 cm^3^. The specimens were subjected to compression tests and loaded either along the gradient (∥) or perpendicular (⊥) to the gradient (Scheme 1b). The specimens were loaded at a speed of 2.5 mm/min while recording the corresponding stress–strain curves. The elastic moduli were calculated from the slope of the initial linear region of the stress–strain curves; the crush strengths were determined at the end of the first linear region. A video extensometer (IMT-CAM018, Imetrum Ltd., Bristol, U.K.) was used to determine the deformation of each layer in the gradient macroporous polymers. For each macroporous polymer and testing direction, at least 5 specimens were characterized.

The elastic moduli *E* and crush strengths σ of macroporous polymers were fitted with their foam density using the power law following the Gibson–Ashby equation
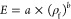
2
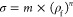
3

Elastic modulus and crush strengths were normalized by the foam density of the macroporous polymers
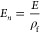
4
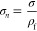
5

Charpy impact tests of macroporous polymers were carried out using a pendulum impact tester (CEAST 9050, Instron GmbH, Germany) equipped with an impact pendulum of 0.5 or 5 J. The macroporous polymers were cut into specimens with dimensions of 1.5 × 1.5 × 7.5 cm^3^ and placed on the testing machine with a span length of 4 cm. The impact pendulum dropped at an angle of 160° to impact the specimens from a direction that was perpendicular to or along the gradient (Scheme 1c). The force was plotted as a function of displacement. The adsorbed energy was calculated by the integration of the entire force–displacement curve.

#### Headform Impact Tests

A 3.5 kg child headform impactor, as specified in the European Automobile Manufacturers Association (ACEA) safety test procedure,^33^ was used to assess the impact mitigation properties of the gradient foams. Headform impactors are used to assess the pedestrian protection afforded by passenger cars. These headforms were introduced in various consumer protection and legislative testing, such as the New Car Assessment Program (NCAP), European regulation 78/2009, and Global Technical Regulation GTR 9. The headform was equipped with triaxial accelerometers (Endevco 7264C) with a measurement range of 500*g*. The resultant acceleration signal is filtered with a low-pass Butterworth filter (CFC 1000). Two injury criteria were extracted from the filtered signal: the head injury criterion (HIC) and the cum3 ms. The HIC considers the regressive correlation between the tolerable acceleration and exposure duration. Most tests require the HIC over a maximum timespan of 36 ms (HIC36) to be less than 1000, which equals the probability of 50% to sustain a serious injury, for example, a concussion with unconsciousness of less than 1 h. A HIC36 of 1000 is, for example, reached, if the head is exposed to a constant acceleration of 60*g*. The second criterion is the cum3 ms, which is the highest acceleration level with a cumulative duration of at least 3 ms, across single or multiple peaks. A common threshold limit for cum3 ms is 80*g*, e.g., in occupant protection. As a third parameter, the coefficient of restitution (COR) was determined, which quantifies the energy loss during the impact. A COR of 0 means total energy loss, indicative of a perfectly plastic collision, while a COR of 1 means no energy loss, i.e., a fully elastic impact.

In addition to the gradient foams (G3 with a density of 190 kg/m^3^), homogeneous reference foams without gradients were also tested, namely, expanded polypropylene (EPP) with a density of 60 kg/m^3^, poly(ethylene terephthalate foam) (PET) with a density of 112 kg/m^3^, poly(vinyl chloride) (PVC) with a density of 97 kg/m^3^, and balsa wood with a density of 130 kg/m^3^. The latter with the wood’s longitudinal (growth) axis parallel to the impact direction. All specimens were 10 × 10 × 2 cm^3^. The hypothesis was that the graded foams provide protection over a wider impact velocity range than homogeneous foams, i.e., that injury criteria are rising to a lesser extent with increasing kinetic energy. To investigate our hypothesis specimens were exposed to 3.1, 3.7, and 4.4 m/s impact tests, each test was repeated 3 times.

## Results and Discussion

**Figure 1:**
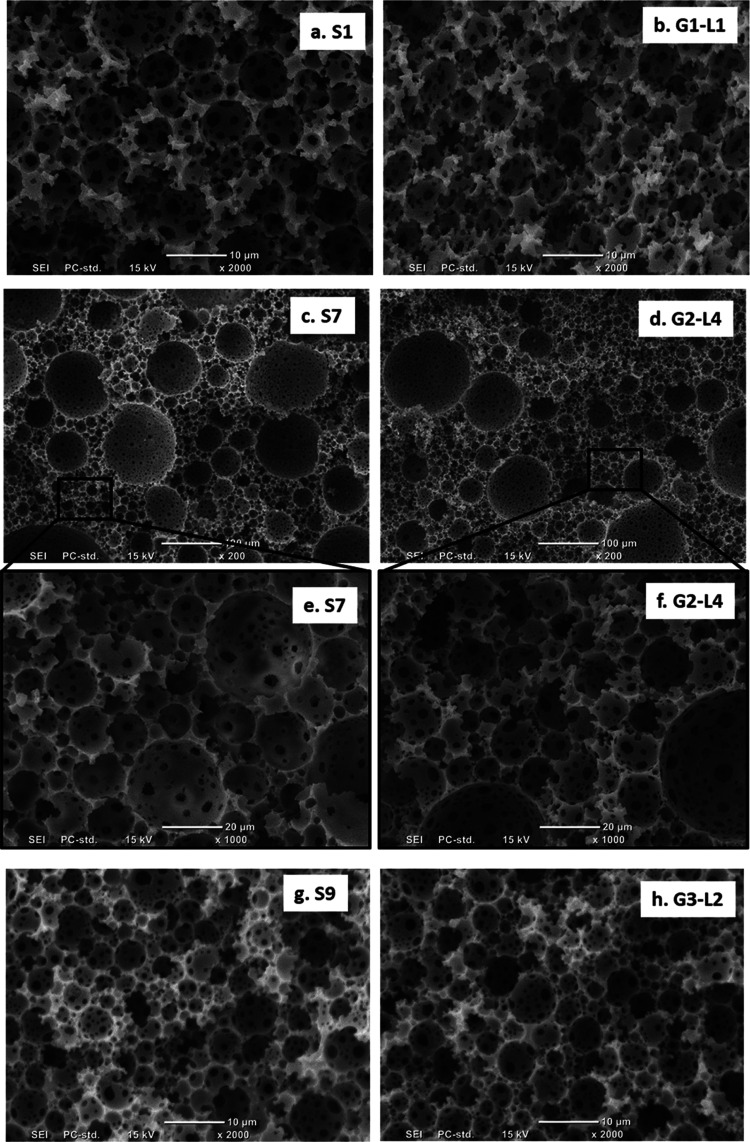
Representative SEM images of emulsion-templated macroporous polymers. (a) S1, homogeneous polyHIPE produced by polymerization of emulsions with an 80 vol % internal phase, stirred at 1000 rpm. (b) G1-L1, first layer (from bottom) in macroporous polymers with gradient foam density G1 (S1 is its control). (c, e) S7, homogeneous polyHIPE, templated using emulsions with an 80 vol % internal phase and stirred at 400 rpm. (d, f) G2-L4, fourth layer (from bottom) in macroporous polymers with gradient pore size G2 (S7 is its control). (g) S9, homogeneous polyHIPE, produced by polymerization of a 1:2 mixture of emulsion templates comprising of an oil phase of St, DVB, and PUDA as monomers (E1) and EHA and PUDA as monomers (E10). (h) G3-L2, second layer (from bottom) in polyHIPE with gradient composition G3 (S9 is its control). The SEM images of all controls (S1–S10) and layers in gradient macroporous polymers (G1-G3) are included in the ESI (Figure S1, ESI).

E1 was formulated containing St, DVB, and PUDA—used as a long chain cross-linker with a flexible backbone to reduce the brittleness of the resulting copolymer^34^ as monomers in their continuous phase and 80% internal phase. Diluting E1 with its own continuous phase resulted in E2–E4 having internal phase ratios reduced to 50%. Casting E1–E4 on top of each other (Table 2), followed by polymerization and purification, resulted in a gradient macroporous poly(St-*co*-DVB-*co*-PUDA) (G1). After purification and drying, G1 did not show any visible deformation or defects in the transition areas between layers, indicating good adhesion between layers. The foam density of each layer of gradient foam G1 from bottom to top (G1-L1 to G1-L4) indeed increased from 0.2 to 0.38 g/cm^3^ (Table 2) and was identical within error to their corresponding controls (S1–S4, Table 2). All layers of G1 possessed the typical pore morphology as common emulsion-templated polymers,^35^ in which spherical pores are interconnected by pore throats (Figures 1 and S1, ESI). The pore and pore throat sizes of the different layers were identical within error (Table 2), which resulted from the fact that the emulsion templates E2–E4 were diluted from E1 and, thus, contained droplets of similar size as E1. As such, we were able to produce a macroporous polymer with gradient foam density, while its composition and average pore size did not vary. When the entire G1 was scanned along the gradient, transition areas between adjacent layers were indistinguishable because of the identical average pore sizes in each layer. However, solid polymer layers were not found in the layer interfaces, indicating that the emulsion templates at these transition regions were stable.

**Table 2:** Summary of Skeletal ρ_s_ and Foam ρ_f_ Densities, Porosity *P*, Pore *d*_p_, and Pore Throat Diameter *d*_pt_ of Homogeneous Macroporous Polymers (S1–S10) and Macroporous Polymers with Gradient Foam Density (G1), Pore Size (G2), and Composition (G3)

	ρ_s_ [g/cm^3^]	ρ_f_ [g/cm^3^]	*P* [%]		ρ_f_ [g/cm^3^]	*d*_p_ [μm]	*d*_pt_ [μm]
S1^a^	1.05 ± 0.03	0.18 ± 0.01	83 ± 1			3.6 ± 2.3	1.2 ± 0.5
S2^a^	1.07 ± 0.02	0.23 ± 0.01	79 ± 1			5.1 ± 1.7	1.3 ± 0.5
S3^a^	1.06 ± 0.01	0.34 ± 0.01	68 ± 1			5.2 ± 2.3	1.3 ± 0.5
S4^a^	1.07 ± 0.01	0.40 ± 0.02	62 ± 2			5.5 ± 2.7	2.4 ± 1.2
S5	1.07 ± 0.01	0.19 ± 0.01	82 ± 1			2.2 ± 1.1	0.8 ± 0.3
S6	1.07 ± 0.01	0.18 ± 0.02	84 ± 1			2.6 ± 1.5	1.0 ± 0.4
S7	1.07 ± 0.01	0.15 ± 0.01	86 ± 1			6.6 ± 8.6	1.6 ± 0.9
S8	1.14 ± 0.01	0.19 ± 0.01	83 ± 1			3.4 ± 1.1	1.0 ± 0.2
S9	1.16 ± 0.01	0.21 ± 0.01	82 ± 1			3.5 ± 1.5	1.1 ± 0.3
S10	1.01 ± 0.01	0.24 ± 0.02	77 ± 1			3.8 ± 2.4	0.9 ± 0.6
G1^a^	1.07 ± 0.02	0.30 ± 0.01	72 ± 1	G1-L1 (S1)^a^	0.20 ± 0.01	4.2 ± 1.7	1.2 ± 0.5
				G1-L2 (S2)	0.23 ± 0.01	4.3 ± 2.6	1.1 ± 0.5
				G1-L3 (S3)	0.33 ± 0.01	3.8 ± 1.7	1.3 ± 0.6
				G1-L4 (S4)	0.38 ± 0.01	4.2 ± 2.1	1.2 ± 0.5
G2	1.07 ± 0.01	0.18 ± 0.01	85 ± 1	G2-L1 (S5)	0.19 ± 0.01	2.4 ± 0.9	0.7 ± 0.3
				G2-L2 (S6)	0.19 ± 0.01	2.8 ± 1.2	1.0 ± 0.4
				G2-L3 (S1)	0.19 ± 0.01	5.4 ± 2.5	1.3 ± 0.6
				G2-L4 (S7)	0.14 ± 0.01	7.5 ± 6.2	1.9 ± 1.0
G3	1.13 ± 0.01	0.19 ± 0.01	83 ± 1	G3-L1 (S10)	0.23 ± 0.01	6.7 ± 1.6	1.4 ± 0.4
				G3-L2 (S9)	0.20 ± 0.01	4.4 ± 1.3	0.9 ± 0.3
				G3-L3 (S8)	0.19 ± 0.01	4.6 ± 1.0	0.8 ± 0.3
				G3-L4 (S1)	0.17 ± 0.01	5.7 ± 1.2	1.4 ± 0.5

a The sample ID in the brackets refers to the corresponding controls of the layer in gradient macroporous polymers.

**Figure 2:**
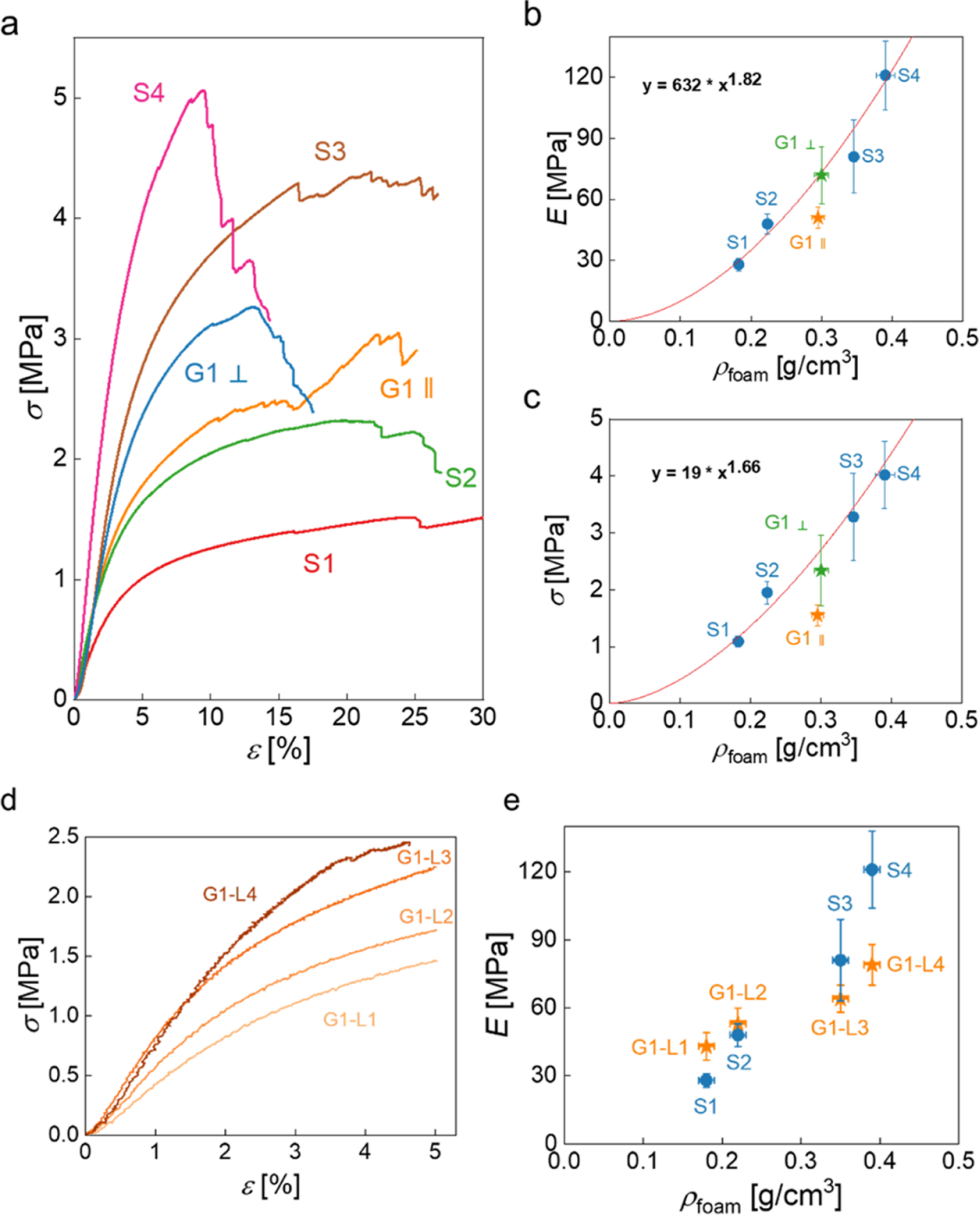
(a) Characteristic compression stress–strain curves of macroporous poly(St-*co*-DVB-*co*-PUDA) with density gradient G1 and four homogeneous macroporous polymers (S1–S4). Elastic moduli *E*_c_ (b) and crush strengths σ_c_ (c) of S1–S4 and G1 as power function of their foam densities ρ_f_. (d) Compressive stress–strain curves of four layers in macroporous poly(St-*co*-DVB-*co*-PUDA) with density gradient G1. (e) The elastic moduli of four layers in G1 compared to their corresponding controls S1–S4.

The stress–strain curve of control S1 comprised a first linear region, followed by a plastic deformation plateau characteristic for porous polymers. With increasing foam density from 0.18 to 0.40 g/cm^3^, S3 and S4 showed more pronounced brittle failure after the first linear region (Figure 2a). The elastic moduli of S1 to S4 increased with increasing foam density from 28 to 121 MPa and the crush strengths from 1.1 to 4.0 MPa. The relationship between elastic moduli and crush strengths and foam density of S1–S4 followed the Gibson–Ashby model (eq 1, Figure 2b,c).^36−38^ When compressing G1 perpendicular to the density gradient (Table 2), the four layers deformed equally and simultaneously as expected, and the global elastic modulus and the crush strength of G1 were close to an average of those of the four individual layers and are described by the Gibson–Ashby model of the controls S1–S4 (Figure 2b,c). During compression of G1 along to the density gradient, we expected that the specimens would fail starting in the weakest layer (G1-L1) and finally in the strongest layer (G1-L4) and show four distinguished stress–strain profiles (similar to ref (39)). However, only a single linear elastic deformation region was observed, followed by failure of the weak layers. Thereafter, the stress increased but was accompanied by small steps of failure. This agreed with the fact that the strong layers carried the load, while cracks propagated into adjacent layers, which caused localized failures in these layers. The propagation of cracks into adjacent layers indicates good adhesion between the layers. The elastic modulus and crush strength of G1 compressed along the density gradient (*E* = 50 MPa and σ = 1.5 MPa) were lower than the Gibson–Ashby fit suggested (Figure 2b,c), which indicates that the deformation indeed occurred primarily in the weakest layers of G1. The elastic modulus of each layer comprising G1 in compression along the density gradient was calculated from stress–strain curves—the strains were measured using a video extensometer (Figure 2d). The elastic moduli of the layers were not significantly different as compared to their controls (Figure 2e). To confirm whether the mechanical behavior of layers comprising G1 is determined by their foam density rather than their location within the specimen, we determined stress–strain curves of four “virtual” layers in a homogeneous control S2 (Figure S2, see ESI). It is clear from the result that the mechanical properties throughout S2 are identical, confirming that the different mechanical properties throughout G1 are solely caused by the density gradient.

In order to produce macroporous polymers with various pore sizes, we formulated HIPE templates containing St, DVB, and PUDA as monomers and 80% internal phase at different stirring speeds (energy input = time × stirring speed) ranging from 400 to 2000 rpm to tune the average droplet size of the HIPEs. These HIPE templates (E5, E6, E1, and E7) were stacked into four layers in a PTFE mold followed by polymerization and purification, resulting in gradient poly(St-*co*-DVB-*co*-PUDA)HIPEs (G2). As anticipated, each layer of polyHIPE had identical foam density (with G2-L4 lower than the other layers; the same situation holds true for its control polyHIPE S7, Table 2). The average pore size of the four layers from bottom to top in G2 increased from 2 to 7 μm (Table 2) and the pore size distribution broadened (Figure S3). The change in the pore size distribution in G2 agreed well with the corresponding controls of every layer. The nonlinear relationship between the stirring speed (energy input) and average pore (droplet) sizes of the polyHIPEs (emulsion templates) (Figures S1 and S3, ESI) was already demonstrated by Tebboth et al.^40^ The different average pore sizes in layers present in G2 allowed transition areas to be distinguished, e.g., G2-L2 and G2-L3 (Figure S4). The morphology of the transition areas between G2-L2 and G2-L3 showed, again, only typical polyHIPE pore structures but not solid polymer layers, supporting our finding that the emulsion templates at the boundary regions were stable during polymerization.

**Figure 3:**
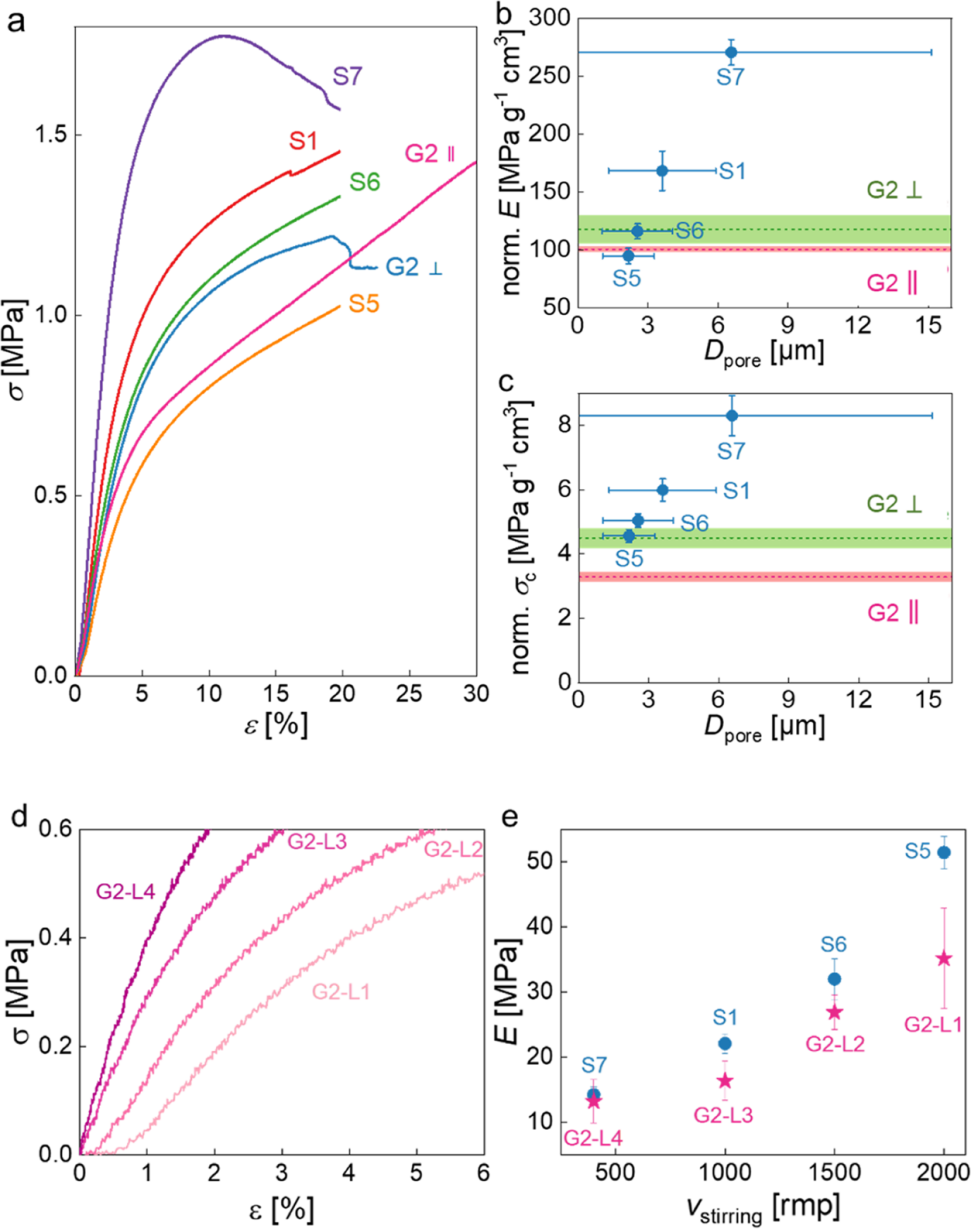
(a) Stress–strain curves of polyHIPEs having a pore size gradient (G2) and corresponding controls with homogeneous pore sizes (S5, S6, S1, and S7). (b and c) Normalized elastic moduli and crush strengths over foam density of polyHIPEs G2, S5, S6, S1, and S7 as a function of their average pore size: values of S5, S6, S1, and S7 are presented by dots with error bars representing standard deviation, while the values of G2 are presented as two lines as its average pore size cannot be projected to the *x*-axis. (d) Compressive stress–strain curves of four layers in macroporous poly(St-*co*-DVB-*co*-PUDA) with pore size gradient (G2). (e) Elastic moduli of four layers in G2 compared to their corresponding controls S5, S6, S1, and S7.

The elastic moduli and crush strengths of the controls S5, S6, S1, and S7 increased with increasing average pore size (Figure 3), which is consistent with the findings reported in the literature.^41,42^ When being compressed perpendicular to the pore size gradient, G2 had a normalized elastic modulus and crush strength close to the average of the controls of the four layers comprising G2, while being compressed along the pore size gradient, G2 showed a normalized elastic modulus and crush strength close to those of the weakest layer (G2-L1, whose control is S5). The elastic moduli of each layer of polyHIPEs, determined using the strains obtained from the video extensometer (Figure 3d), are in agreement with those of the corresponding controls (Figure 3e).

Emulsion templates containing St, DVB, and PUDA as monomers (E1) and EHA and PUDA as monomers (E10) were formulated to be polymerized to produce stiff (S1) and flexible polyHIPEs (S10). E1 and E10 were also mixed in various ratios of 100:0, 66:33, 33:66, and 0:100; these emulsion templates (E1, E8, E9, and E10) were stacked and polymerized to produce a polyHIPE (G3) with gradient chemical composition and stiffness. Both pore sizes and porosity of the layers in G3 were in a good agreement with their corresponding controls (Table 2). The pore sizes of each layer in G3 were identical within the error, while the foam density of G3-L1 (and its control, S10) was slightly higher than the other layers; this was because of the slight shrinkage due to the flexibility of the poly(EHA-*co*-PUDA)HIPE.

**Figure 4:**
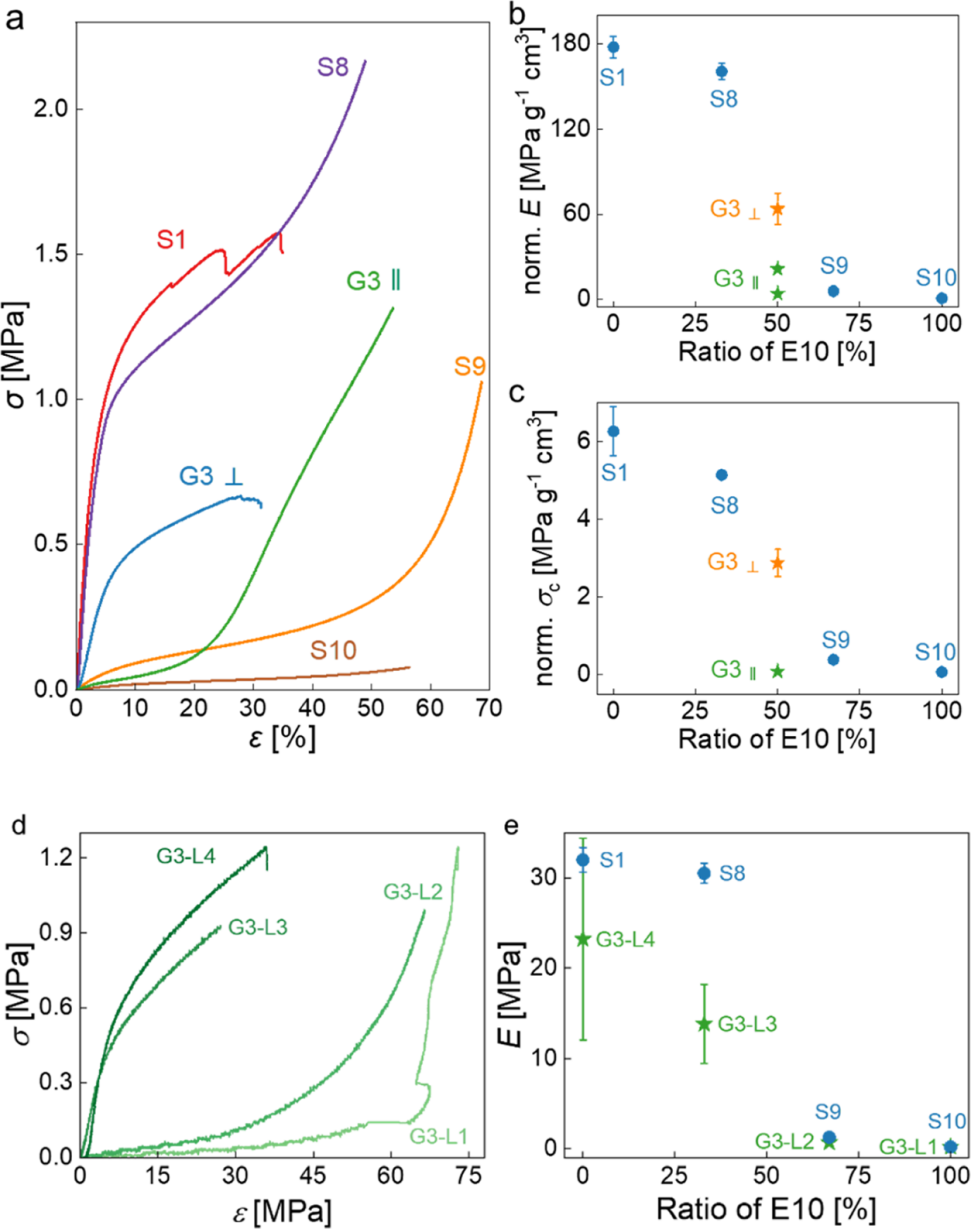
(a) Stress–strain curves of polyHIPEs having a composition gradient (G3) and homogeneous compositions (S1, S8, S9, and S10). (b, c) Normalized elastic moduli and crush strengths over foam density of polyHIPEs G3, S1, S8, S9, and S10 as a function of the composition of emulsion templates. (d) Compressive stress–strain curves of the four layers comprising macroporous poly(St-*co*-DVB-*co*-PUDA) with composition gradient (G3). (e) Elastic moduli of the four layers in G3 compared to their corresponding controls S1, S8, S9, and S10.

Although the mixing ratio of the primary emulsions E1 and E10 was changed linearly, after polymerization and purification the elastic moduli of S1, S8, S9, and S10 did not change in a linear fashion: S8 had a similar modulus to the stiff S1, while S9 exhibited elastomeric behavior close to S10 (Figure 4). This was caused by nonhomogenous mixing of the primary emulsion templates E1 and E10; we blended emulsions but did not re-emulsify them, resulting in a heterogeneous chemical composition, which was already decribed in the literature.^30^ After polymerization, the major volume phase dominated the corresponding mechanical properties of the polyHIPEs. G3 with composition gradient showed three linear regions during compression along the gradient (Figure 4a). The initial linear region represented the elastic deformation of the flexible layer in G3, followed by a second linear region caused by the nonelastic deformation of this flexible layer. The normalized elastic modulus of the third linear region was 25 MPa g^–1^cm^3^, which was too low to represent the deformation of the stiff layers. Therefore, the third linear region was considered to be a combination of the elastic deformation of the stiff layers (G3-L3 and G3-L4) and the densification of the flexible layers (G3-L1 and G3-L2). The elastic moduli of G3-L1 and G3-L2 were determined to be similar to those of their controls (Figure 4e). However, the moduli of G3-L3 and G3-L4 were lower than their corresponding controls and had large standard deviations. During the compression of G3 along the gradient, L1 and L2 deformed massively due to their low moduli, while L3 and L4 underwent only small deformations, which resulted in strains with large deviation determined using a video extensometer.

**Figure 5:**
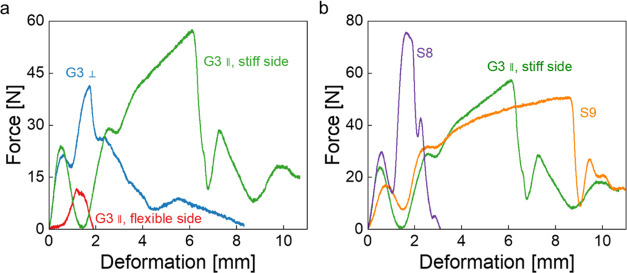
(a) Force–deformation curves of poly(St-*co*-DVB-*co*-PUDA) with gradient composition G3 upon impact loading in three directions: stiff and flexible layers exposed to the load as well as the side perpendicular to the gradient. (b) Force–deformation curves of poly(St-*co*-DVB-*co*-PUDA) with homogeneous (S8 and S9) and gradient composition (G3) upon impact on the stiff layer.

Charpy impact tests were carried out on G3, S1, S8, and S9. It is clear that G3 had significantly better impact resistance, assessed by the high impact peak load and absorbed energy when impact tests were performed on the stiff layer exposed rather than on the flexible layer exposed or on the side perpendicular to the gradient (Figure 5a and Table S1). Based on the fact that S1 underperformed as compared to S8, while S10 was too flexible to be broken under impact, S8 and S9 were used as benchmark to evaluate the impact performance of G3. S8 failed in a brittle manner upon impact, characterized by short deformation upon failure; its impact peak load reached 76 N. S9 had a lower impact peak load of 51 N than S8 but absorbed 351 mJ energy. Also, S9 had a deformation upon failure of larger than 10 mm. The impact performance of G3 fell well between S8 and S9: G3 had reduced impact peak load (62 N) but gained energy adsorption (286 mJ) close to that of S9 and a larger deformation upon failure (Figure 5b and Table S1).

**Figure 6:**
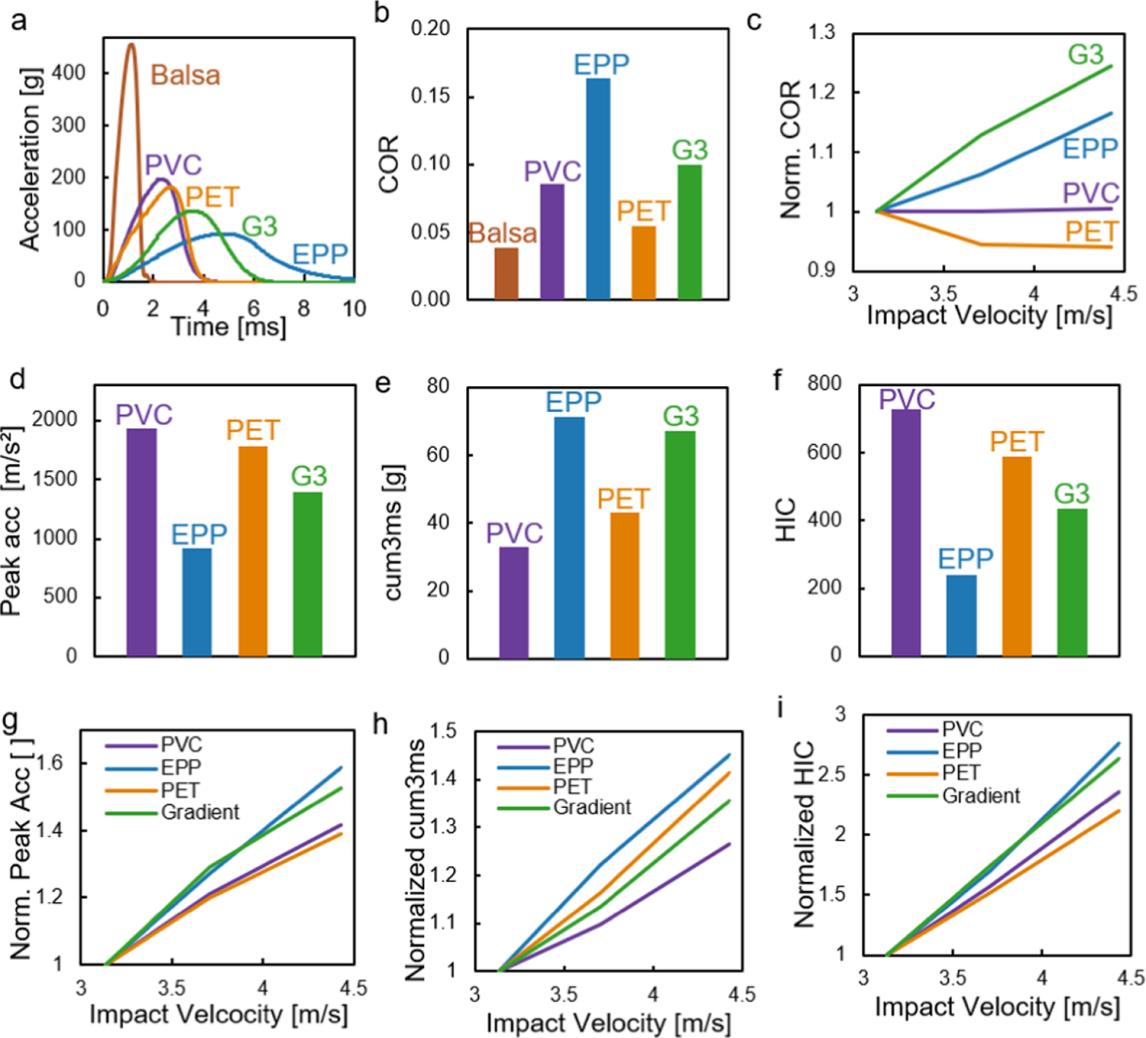
(a) Acceleration–time curves of drop-impact tests (3.1 m/s). (b) Coefficient of restitution (COR (3.1 m/s)). (c) COR over velocity, normalized to 3.1 m/s. (d) Peak acceleration (3.1 m/s). (e) Cumulative 3 ms (3.1 m/s). (f) Head injury criterion (3.1 m/s). (g) Peak acceleration over velocity, normalized to 3.1 m/s. (h) Cumulative 3 ms value over velocity, normalized to 3.1 m/s, i.e., head injury criterion over velocity, normalized to 3.1 m/s. All curves show average values.

The acceleration–time curve recorded during headform impact tests showed that the gradient foam (G3) was qualitatively similar to PVC, with a subtle difference, namely, a short primary phase of about 1 ms with a lower acceleration gradient, i.e., jerk. Both PVC and G3 showed an almost linear unloading phase, while EPP exhibited a regressive behavior in unloading (Figure 6a). The steepest unloading curve was obtained with Balsa and PET, which is also reflected by their low COR (Figure 6b). Similar to PVC, G3 also provided a low COR of only 0.1. For PVC foams the COR remained constant with increasing velocity, elastic rebound increased with velocity for G3 (Figure 6c). G3 had the second lowest peak acceleration (Figure 6d), it returned, however, the second highest cumulative 3 ms value because of its wide apex. The HIC value of G3 was markedly higher than that obtained for EPP but also lower than that of PVC foams (Figure 6f). None of the synthetic foams exceeded the critical HIC of 1000 nor the critical cum3 ms value of 80*g*. Balsa wood, which is not shown in the subfigures c through i for clarity, returned a high critical HIC value of 2650. The change of peak acceleration, cum3 ms, and HIC with velocity (Figure 6,g through (i)) revealed similar, almost linear behavior in all foams under study.

The headform impact test showed that G3 is a reasonable impact absorber, returning injury criteria and peak values in the range of other frequently used polymer foams in impact energy management. We found, however, no evidence to support the hypothesis that gradient foams would provide benefits over a wider range of impact velocities (Figure 6c,g–i). The benefits of gradient over homogeneous foams for impact energy management and injury reduction are controversially discussed in the literature: Klug et al.^43^ could not show a tangible benefit of gradient foams in oblique helmet impact tests (combined translational and rotational loading) in terms of head injury criteria. Cui et al.^5^ showed that the benefit of gradient foams in terms of peak acceleration (in translational loading) exists only in a narrow velocity range (more than 30% reduction). Outside this sweet spot, gradient foams returned slightly higher peak accelerations (∼10%). The highest benefit in that study was obtained with a cubic or quadratic (and not with a linear) material gradient, combined with a high density offset (ΔΔ = 40 kg/m^3^). Zhang and Zhang^44^ studied functional gradient materials with increasing and decreasing gradients in sphere impact tests. While the qualitative behavior of uniform and linearly increasing gradients with respect to the force–displacement behavior remained unchanged over the investigated velocity range (10–35 m/s), the behavior of the foams with decreasing density changed with velocity from a linear to an undulating curve shape (once the struck side layer cracked and busted). As only a limited number of G3 samples were available for headform testing in this present study, it was not possible to investigate the behavior at higher speeds or the impact on the stiff side. It can be concluded that gradient foams could show benefits in terms of injury risk mitigation when the expected impact velocity range is narrow, which is for example the case in children’s bicycle handlebar guards. Gradient foams might have a benefit in the functional integration of outer hardshell, impact liner and comfort liner in future helmet designs.^45^

**Figure 7:**
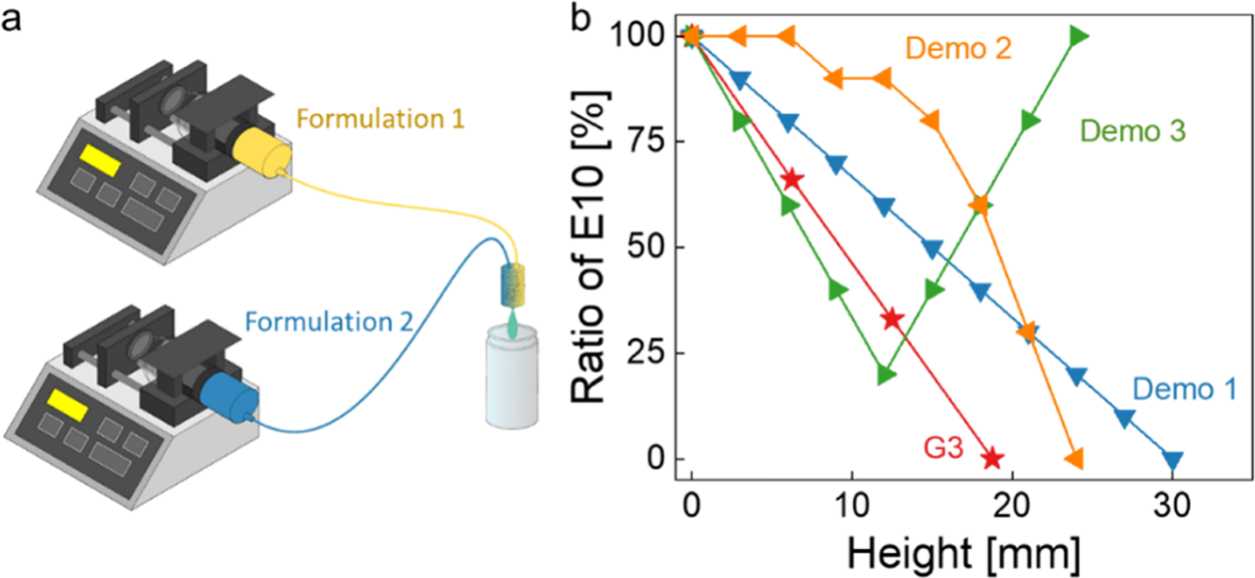
(a) Two syringe pumps used for dispensing two liquid formulations (e.g., two emulsion templates) at controlled ratios into a mixing chamber. After the static mixing, the emulsion was dispensed into a glass vial. (b) Two-syringe setup for preparing emulsion templates with gradient composition: the volume ratio of E10 was plotted as a function of the height of the emulsion templates.

The polyHIPEs with a four-layer stepwise structure highlight the possibility to produce a well-controlled tunable structure exhibiting gradient properties using only two initial formulations; yet manually stacking emulsion templates is too labor intensive to produce true gradient polyHIPEs. To tackle this challenge, we used two syringe pumps to dispense emulsion templates (E1 and E10) at desired volumes through a static mixing unit (Figure 7a). The mixed emulsion templates were extruded into a glass vial to create a composition gradient in the height direction (SI Video 1). The composition gradients throughout the structure were controlled to be linear (Demo 1), curved (Demo 2), and bidirectional (Demo 3) with finer changing steps (as compared to G3, Figure 7b). Demo 3 was polymerized from a bidirectional emulsion composition (e.g., E10-E1-E10). When being loaded from top at a speed of 1 mm/min, the bidirectional gradient polyHIPE did show as expected: the flexible layers at the top and bottom of Demo 3 were more compressed, while the stiff middle layer was less compressed (Figure S5, ESI). With our progress on the production of finer gradient polyHIPEs, true gradient polyHIPEs with an even smoother transition between property steps have yet to be produced. If dual-dispensing systems, which are already commercially available, were used to control the dispensing volume of the two emulsions automatically, true gradient polyHIPEs can be produced. Furthermore, when a dynamic stirrer will be installed in the mixing chamber, changing the droplet size of emulsions and thus the pore size of the resulting polyHIPEs will also be enabled, which would provide a high degree of freedom to map different gradient properties in porous polymers.

## Conclusions

Macroporous polymers with density, pore structure, and composition gradient have been produced by stacking and polymerizing emulsion templates of various formulations. The liquid nature of emulsion templates allowed the blending of two starting (emulsion template) formulations to generate a number of different emulsion templates. We showed that the polymerization of the created gradient emulsion template resulted in macroporous polymers with individually controlled density, pore size, and polymer composition gradients while keeping the other two unaffected. The mechanical properties of each layer in the gradient foam corresponded to their respective controls. The weakest layers dominated the modulus and strengths of the gradient foams when compressing along the gradient.

PolyHIPEs with composition and stiffness gradient showed promising impact resistance, as the foam inherited high peak loads from the stiff layers and energy adsorption and deformation capability from the flexible layers. Headform impact tests, however, did not show a clear advantage of the gradient foams in wearer protection as compared to homogeneous reference foams; future work will focus on developing the gradient foams into functional structures, e.g., providing impact protection and wear comfort, for body protection applications, e.g., helmets.

We demonstrated dual dispensing and simultaneous blending of two very different emulsion formulations, which enables to gradually change template composition. We could show that after polymerization and purification, macroporous materials with fine bidirectional composition gradients can be produced. Our work demonstrated a facile approach toward the realization of true gradient macroporous polymers with a high degree of design freedom.

## Supporting Information Available

Pore morphology of homogeneous and gradient macroporous polymers. Stress–strain curves of virtual layers of homogeneous macroporous polymers. Pore size distribution of polyHIPEs. Morphology of the transition area in gradient macroporous polymers. Impact properties of homogeneous and gradient polyHIPEs. Compression on demonstration polyHIPEs having a bidirectional composition gradient (PDF)
Video showing the preparation of gradient emulsion templates using two syringe pumps dispensing emulsion templates of different compositions (MP4)
Video showing the behaviour of a bi-directional gradient polyHIPE (Demo 3) under compressive loading and unloading (MP4)
