# Supplementary material for: Functionally Gradient Macroporous Polymers: Emulsion Templating Offers Control over Density, Pore Morphology, and Composition
Source: ACS Appl Polym Mater. 2024 Apr 24;6(9):5150–62. doi: 10.1021/acsapm.4c00261 (PMC11091853; doi:10.1021/acsapm.4c00261)
Supplement: Supplementary file 1 — ap4c00261_si_001.pdf [file ap4c00261_si_001.pdf]

Supporting information of:

Functionally Gradient Macroporous Polymers: Emulsion templating offers control over Density, Pore Morphology and Composition

Yufeng Xu[**, 1], Le Tang[**, 1], Chanokporn Nok-iangthong[1], Markus Wagner[2], Georg Baumann[2], Florian Feist[2], Alexander Bismarck[*,1,3], Qixiang Jiang[*,1]

[1] Institute of Material Chemistry and Research, Faculty of Chemistry, University of Vienna, Währinger Strasse 42, 1090, Vienna
[2] Institute for Vehicle Safety, Graz University of Technology, Inffeldgasse 13 VI, 8010 Graz
[3] Department of Chemical Engineering, Imperial College London, South Kensington Campus, London, SW7 2AZ, UK.
[*]Corresponding authors: Qixiang.jiang@univie.ac.at; alexander.bismarck@univie.ac.at
[**]Y. Xu and L. Tang contributed equally in conducting the experiments and writing and are both credited as first author.

Gradient density

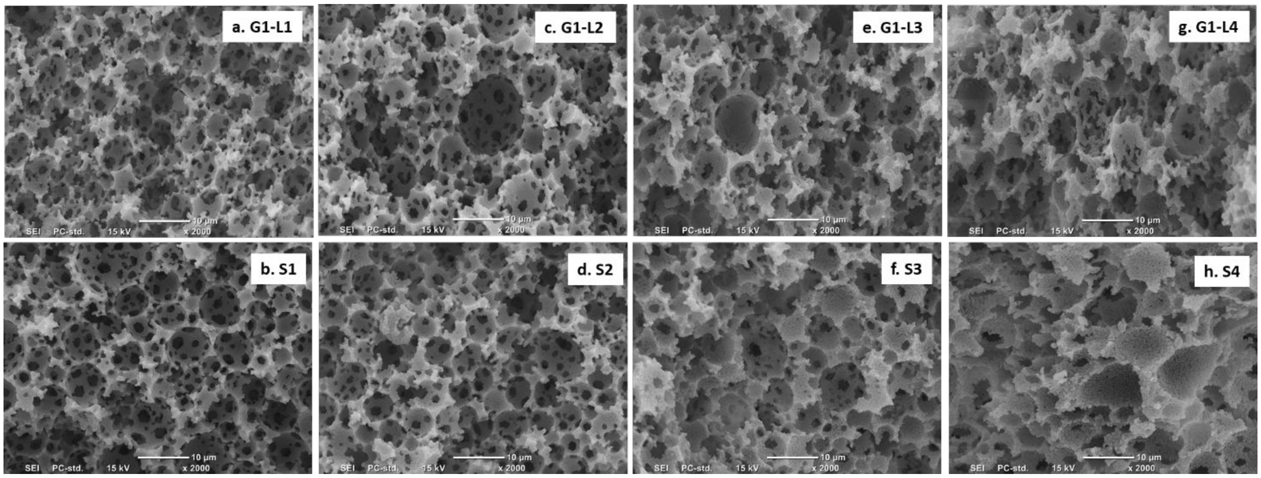

Gradient pore size

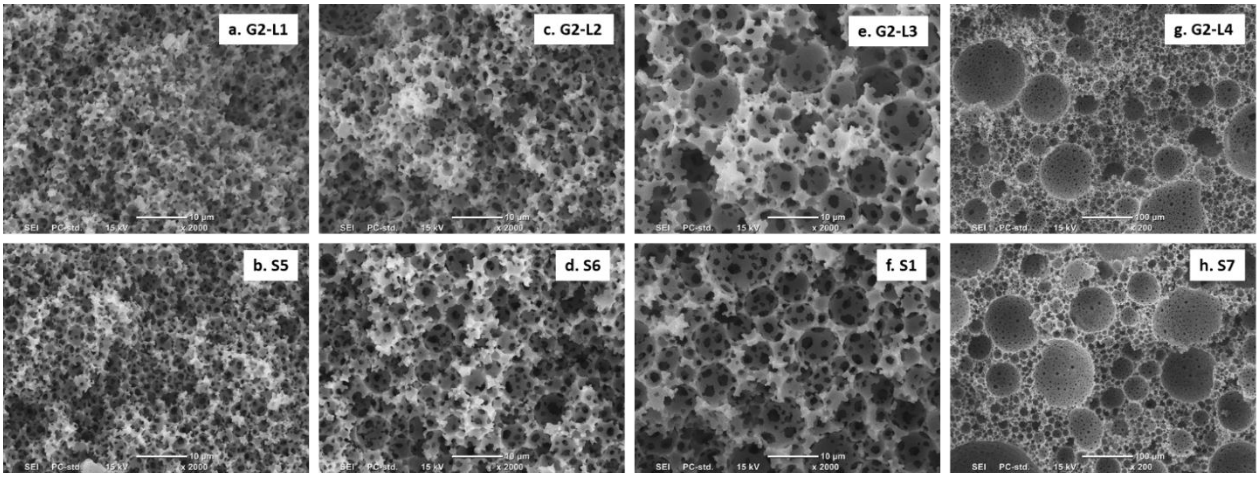

Gradient compositions

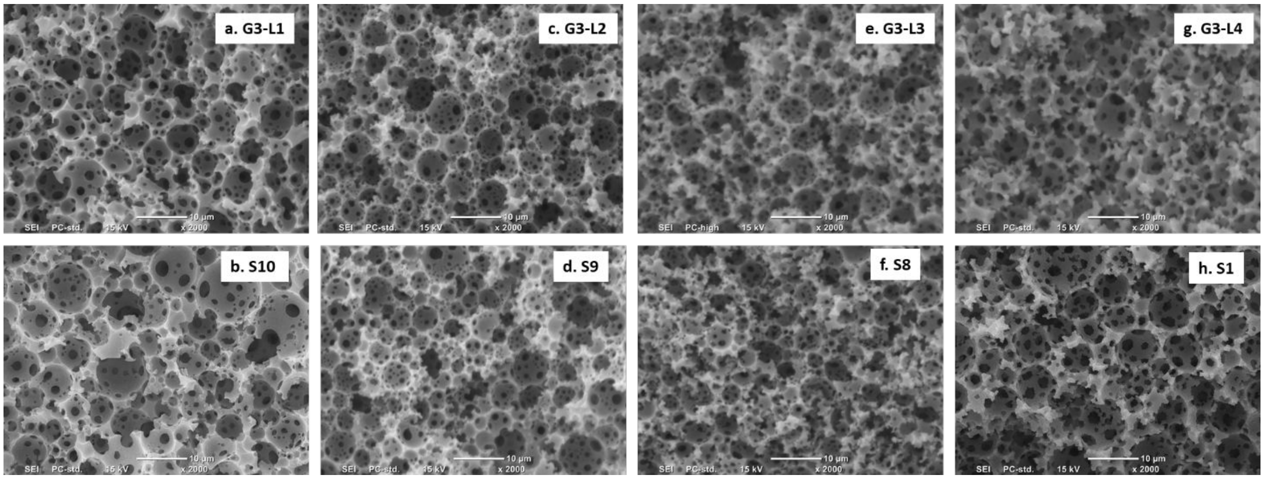

Figure S1 Representative SEM images of emulsion templated macroporous polymers. The SEM images showed the pore morphology of each layer in gradient macroporous polymers that was controllable by its corresponding controls.

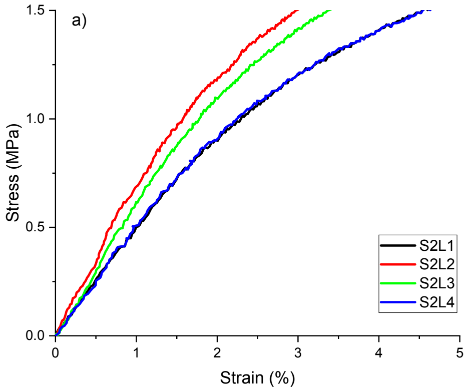

Figure S2 Compression stress-strain curves of “virtual” layers in a homogenous control S2.

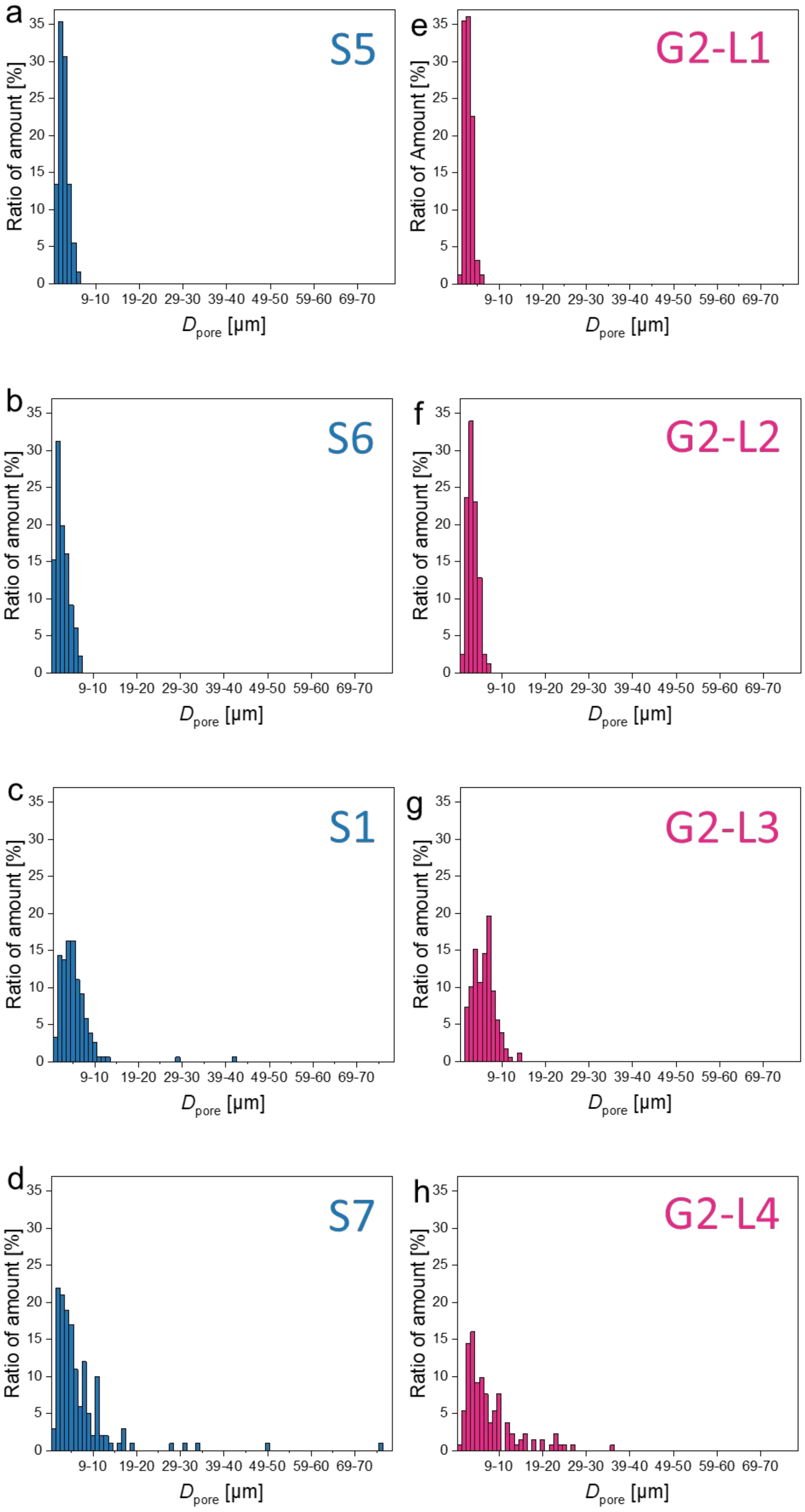
a
S5
b
S6
c
S1
d
S7
e
G2-L1
f
G2-L2
g
G2-L3
h
G2-L4
Ratio of amount [%]
Ratio of Amount [%]
0 5 10 15 20 25 30 35
9-10 19-20 29-30 39-40 49-50 59-60 69-70
D_{pore} [μm]

Figure S3. Pore size distribution of emulsion templated macroporous poly(St-co-DVB-co-PUDA) having pore size gradient and homogenous pore sizes. a-d. the four layers of G2, templated by HIPEs prepared by stirring at 2000, 1500, 1000 and 400 rpm. e-h. the control polyHIPEs templated by emulsions prepared by stirring at 2000, 1500, 1000 and 400 rpm.

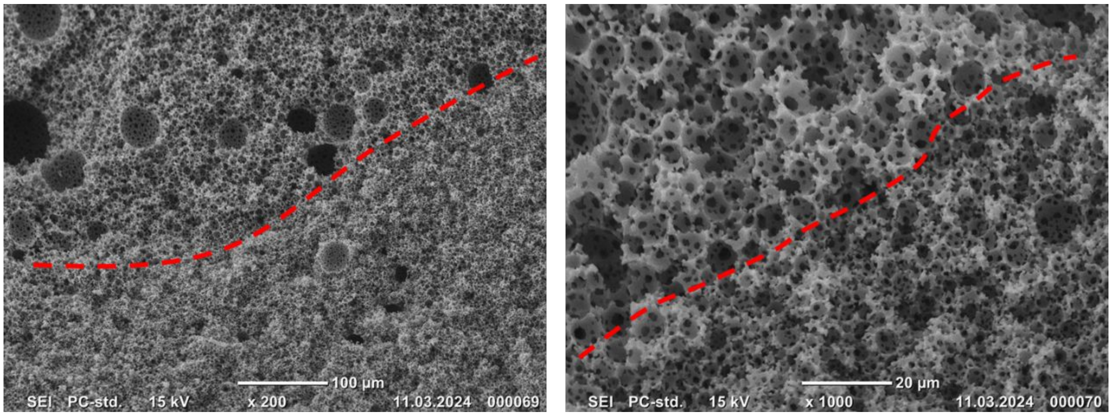

Figure S4. Transition area between G2-L2 and G2-L3 (low and high magnifications). The transition area distinguished layers of macroporous polymers because their different average pore sizes. There is no solid polymer layer at the transition area, indicating there was no phase separation in the transition region of the emulsion templates. Such layer transitions are present in all gradient porous polymers G1-G3. However, transition areas in G1 and G3 were indistinguishable due to the similar average pore sizes of adjacent layers in macroporous polymers. The only distinguishable transition areas were G2-L2 / G2-L3 as shown in Figure S4 and G2-L3 / G2-L4.

Table S1. Peak load and total absorbed energy of macroporous polymers with composition gradient G3 from impact tests performed on stiff, flexible layers and side perpendicular to the gradient and homogenous macroporous polymers S1, S8 and S9

	Peak load [N]	Total absorbed energy [mJ]
G3 ǁ, flexible side	14 ± 5	14 ± 8
G3 ⊥	36 ± 5	103 ± 5
G3 ǁ, stiff side	62 ± 8	286 ± 35
S1	58 ± 11	53 ± 10
S8	76 ± 1	97 ± 5
S9	51 ± 6	351 ± 53

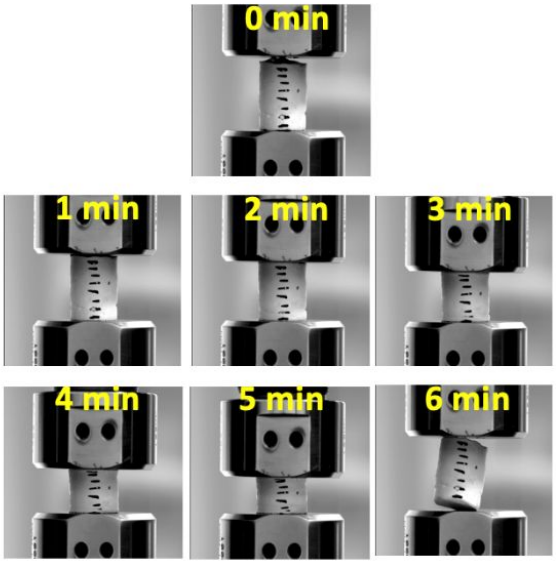

Figure S5. Demonstrative polyHIPE with a bi-directional composition was subjected to a compression test. The test was video recorded (ESI V2).

ESI V1: Video showing the preparation of gradient emulsion templates using two syringe pumps dispensing emulsion templates of different compositions.

ESI V2: Video showing the behaviour of a bi-directional gradient polyHIPE (Demo 3) under compressive loading and unloading.
